# Perceived outcomes of periacetabular osteotomy: a social media analysis

**DOI:** 10.1302/2633-1462.51.BJO-2023-0093.R1

**Published:** 2024-01-19

**Authors:** Ryan Bialaszewski, John Gaddis, Bretton Laboret, Elizabeth Bergman, Edward P. Mulligan, Jenny LaCross, Adina Stewart, Joel Wells

**Affiliations:** 1 The University of Texas Rio Grande Valley School of Medicine, Edinburg, Texas, USA; 2 University of Texas Southwestern Medical Center, Dallas, Texas, USA; 3 Texas Woman's University, Houston, Texas, USA; 4 Tufts University School of Medicine, Boston, Massachusetts, USA; 5 Texas Woman's University, Denton, Texas, USA; 6 Baylor Scott & White Hip Preservation Center and Comprehensive Hip Center, McKinney, Texas, USA

**Keywords:** Periacetabular osteotomy, PAO, Outcomes, Social media, Periacetabular Osteotomy, physicians, postoperative pain, surgical outcomes, symptomatic acetabular dysplasia, patient-reported outcome measures, physical therapy, orthopaedic surgeons, surgical complications, orthopaedic surgery

## Abstract

**Aims:**

Social media is a popular resource for patients seeking medical information and sharing experiences. periacetabular osteotomy (PAO) is the gold-standard treatment for symptomatic acetabular dysplasia with good long-term outcomes. However, little is known regarding the perceived outcomes of PAO on social media. The aims of this study were to describe the perceived outcomes following PAO using three social media platforms: Facebook, Instagram, and X (formerly known as Twitter).

**Methods:**

Facebook, Instagram, and X posts were retrospectively collected from 1 February 2023. Facebook posts were collected from the two most populated interest groups: “periacetabular osteotomy” and “PAO Australia.” Instagram and X posts were queried using the most popular hashtags: #PAOwarrior, #periacetabularosteotomy, #periacetabularosteotomyrecovery, #PAOsurgery, and #PAOrecovery. Posts were assessed for demographic data (sex, race, location), perspective (patient, physician, professional organization, industry), timing (preoperative vs postoperative), and perceived outcome (positive, negative, neutral).

**Results:**

A total of 1,054 Facebook posts, 1,003 Instagram posts, and 502 X posts were consecutively assessed from 887 unique authors. The majority (63.3%) of these posts were from patients in the postoperative period, with a median of 84 days postoperatively (interquartile range 20 to 275). The longest follow-up timeframe postoperatively was 20 years. Regarding perceived outcomes, 52.8% expressed satisfaction, 39.7% held neutral opinions, and 7.5% were dissatisfied. Most dissatisfied patients (50.9%) reported pain (chronic or uncontrolled acute) as an attributing factor.

**Conclusion:**

Most PAO-perceived surgical outcomes on social media had a positive tone. Findings also indicate that a small percentage of patients reported negative perceived outcomes. However, dissatisfaction with PAO primarily stemmed from postoperative pain. Social media posts from other sources (physicians, hospitals, professional organizations, etc.) trend towards neutrality. Healthcare providers must consider the social media narratives of patients following PAO, as they may reveal additional outcome expectations and help improve patient-centred care, create informed decision-making, and optimize treatment outcomes.

Cite this article: *Bone Jt Open* 2024;5(1):53–59.

## Introduction

Periacetabular osteotomy (PAO) is a juxta-acetabular osteotomy and is the most commonly used procedure for the majority of adults with dysplastic hips, with good outcomes in the short-, intermediate, and long-term timeframes.^1-6^ However, given the complexity and potential complications following PAO, patient perspectives and experiences following this intervention are of great interest to orthopaedic surgeons. Patient perceptions have traditionally been assessed through surveys and clinical follow-up encounters, providing valuable but limited insights.

The emergence of social media has revolutionized how people communicate and seek information. This includes patients with musculoskeletal conditions who use social media to share their experiences and obtain information about their health condition. Analysis of this underutilized and valuable source of information may help improve patient-centred care, inform decision-making, and optimize treatment outcomes. Prior studies have examined the use of social media platforms to understand perceptions of various orthopaedic and other surgical interventions, including, but not limited to, breast reconstruction, brain aneurysm repair, hip arthroscopy, and migraine surgery.^7-20^ To our knowledge, no prior studies have examined the use of social media to evaluate the perceived outcomes of PAO. Therefore, the purpose of this study is to conduct the first analysis of social media posts to investigate patient-perceived outcomes related to PAO, and to identify factors associated with a negative perceived outcome. We hypothesized that a majority of perceived outcomes following PAO would be positive, and any negative perceived outcomes were more likely to be associated with a complication.

## Methods

Institutional review board approval was not required for this study as it involved secondary analysis of publicly available social media posts. A retrospective review of social media posts was conducted on three popular platforms: Facebook, Instagram, and X (formerly known as Twitter). Data collection was consecutive and spanned from 11/23/2011 to 02/01/2023, intending to obtain 1,000 unique posts from each platform. This sample size was chosen to ensure a large database for analysis, and to surpass the scope of prior studies in healthcare-related social media analyses.

A total of 1,054 Facebook posts were collected, dating back to November 2021; 1,003 Instagram posts were collected from June 2021. For X, post collection was concluded after 12 years (November 2011) with a total of 502 posts. The decision to halt the retrospective collection of X posts after this duration was based on the belief that social media usage and reported outcomes before this period might not be as relevant to currently perceived outcomes. Usernames and names of authors were cross-referenced between social media sites to the best of our ability to determine the number of unique authors (887).

The collection of Facebook posts focused on the two most populated interest groups, “Periacetabular Osteotomy” and “Periacetabular Osteotomy Australia,” with 8,800 and 431 members, respectively. Access to the private Facebook group “Periacetabular Osteotomy (PAO) UK Based Group” with 1,100 members was not granted. Instagram and X posts were identified using popular hashtags related to PAO based on their frequency of use. These hashtags included: #PAOwarrior (11,300), #periacetabularosteotomy (8,800), #PAOsurgery (7,800), #PAOrecovery (3,500), and #periacetabularosteotomyrecovery (643) (Table I).

**Table I.** Post origin by social media platform, group, and/or hashtag use frequency.

Post origin	Facebook	Instagram	X
**Group, n**			
Periacetabular Osteotomy	948		
Periacetabular Osteotomy Australia	106		
Hashtag frequency, n*			
#PAOwarrior		748	199
#periacetabularosteotomy		387	153
#periacetabularosteotomyrecovery		39	0
#PAOsugery		277	184
#PAOrecovery		188	31
**Total posts, n**	1,054	1,003	502

* Many posts on Instagram and X used multiple hashtags; therefore, the frequency of hashtag utlization does not reflect the total number of posts collected.

Posts were assessed for several key factors: demographic data (sex and race), location (if provided), perspective (patient, family/friend, physician, hospital or physical therapy group, professional organization, news media, industry), timing (preoperative, postoperative, nonoperative), and perceived outcome (positive, negative, neutral). Distinguishing between preoperative or nonoperative posts, and any reported concomitant surgeries, were determined through inferred references to surgical evaluations or dates. Inclusion criteria comprised posts related to the PAO within the postoperative timeframe and written in English. Posts in languages other than English were excluded to minimize interpretation bias.

All posts related to a perceived outcome were identified and assigned to one of three categories; positive, negative, or neutral. Posts were assigned a category based on the explicit or inferenced expression in the text accompanied by any media regarding attitude toward the PAO. For example, a post that said, “I’m so glad I had my PAO,” with a picture of the patient smiling and providing a ‘thumbs up’ gesture would be assigned as positive. A post that mentioned, “My surgery has led to me being in even more pain,” would be categorized as negative, and a post that states, “I am now eight weeks out from surgery,” without further context, was categorized as neutral.

### Statistical analysis

To ensure posts were categorized consistently and to further minimize interpretation bias, each author reviewed an additional 20 posts collected from other authors with high agreeance (86.7% interobserver agreement when analyzing outcome categorization). Non-parametric chi-squared tests were performed to determine significance when applicable with SPSS Statistics for Mac, version 28 (IBM, USA). The significance threshold was set at 0.05.

## Results

**Fig. 1:**
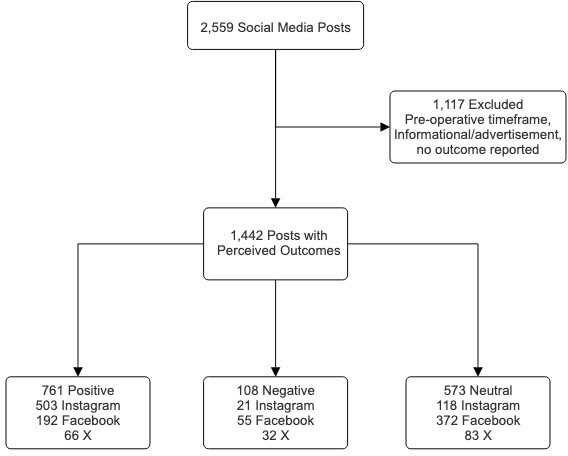
Flowchart showing the study population, reasons for exclusion, and outcomes.

Overall, 87.0% of the posts were from the patient’s perspective, 6.1% of posts were from family or friends, 3.6% of the posts were attributed to professional organizations, 1.9% were from hospitals or physical therapy groups, 0.8% from physicians, and 0.7% from news media or industry. Females constituted 89.7% of the posts, whereas males accounted for 4.0%. Hospitals, physical therapy groups, professional organizations, news media, and industry comprised the other 6.3% in which sex was not categorized. Out of the 2,559 posts collected, 1,684 (65.8%) were from the postoperative timeframe (Figure 1). The median follow-up timeframe for posts referencing a surgery date was 84 days postoperatively (interquartile range (IQR) 20 to 275). The longest follow-up timeframe postoperatively was 20 years. The remaining 875 posts were preoperative (498 posts, 19.5%) or nonoperative (377 posts, 14.7%) (Table II). Posts that did not indicate a surgical date or have an explicitly stated postoperative timeframe were not included in the follow-up timeframe calculations.

**Table II.** Baseline characteristics of social media post authors.

Baseline characteristic	Instagram	Facebook	X
Unique author, n	195	530	162
**Perspective, n (%)**			
Patient	868 (86.5)	921 (87.4)	436 (86.9)
Family or friend	8 (0.8)	129 (12.2)	20 (4.0)
Physician	0 (0)	0 (0)	20 (4.0)
Hopsital or physical therapy group	45 (4.5)	0 (0)	3 (0.6)
Professional organization	79 (7.9)	1 (0.1)	11 (2.2)
News media	0 (0)	0 (0)	2 (0.4)
Industry	3 (0.3)	3 (0.3)	10 (2.0)
**Sex, n (%)**			
Female	851 (84.8)	1,018 (96.6)	426 (84.9)
Male	36 (3.6)	36 (3.4)	30 (5.9)
Unknown	116 (11.6)	0 (0)	46 (9.2)
**Ethnicity, n (%)**			
American Indian or Alaska Native	1 (0.1)	2 (0.2)	0 (0)
Asian	59 (5.9)	19 (1.8)	10 (2.0)
Black or African American	38 (3.8)	15 (1.4)	10 (2.0)
Hispanic or Latino	6 (0.6)	108 (10.2)	23 (4.5)
Native Hawaiian or Other Pacific Islander	1 (0.1)	7 (0.7)	0 (0)
White	682 (68.0)	867 (82.3)	409 (81.6)
Other or Unknown	216 (21.5)	36 (3.4)	50 (10.0)

Of the 1,442 posts related to a perceived outcome, 761 (52.8%) were categorized as positive. Instagram accounted for two-thirds of all posts with a positive perceived outcome (503 posts; 66.0%) and was more likely to see positive perceived outcomes when compared to Facebook and X (78.3% vs 32.3%, p < 0.001). Notably, 573 of all posts (39.7%) were categorized as having a neutral perceived outcome and were more likely to be reported on Facebook when compared to Instagram and X (60.0% vs 24.4%, p < 0.001) (Table III).

**Table III.** Perceived outcome categories by social media platform.

Social media platform	Positive, n (%)	Neutral, n (%)	Negative, n (%)
Instagram	503 (78.3)	118 (18.4)	21 (3.3)
Facebook	192 (31.0)	372 (60.1)	55 (8.9)
X	66 (36.5)	83 (45.9)	32 (17.7)

The remaining 108 posts (7.5%) were categorized as having a negative perceived outcome, with X being more likely to express such an outcome when compared to Facebook and Instagram (17.7% vs 6.0%, p < 0.001). Among these posts, the majority (50.9%, 55 posts) reported pain (chronic or uncontrolled acute) as an attributing factor. Those who reported any complication were more likely to report a negative perceived outcome (75.9% vs 17.7%, p < 0.001) (Table IV). Furthermore, posts that reported a concomitant surgery (arthroscopy, femoral osteotomy, bilateral PAOs, or other additional surgery) were not more likely to report a negative outcome when compared to those who did not have a concomitant surgery (14.8% vs 14.3%, p = 0.887) (Table V). Additionally, our data showed that posts with positive outcomes consistently received higher average numbers of comments and likes across all social media platforms than negative outcome posts, possibly due to an inherent bias to post positive outcomes (Table VI).

**Table IV.** Factors associated with negative perceived outcome.

Social media platform	Negative posts reporting pain, n (%)	Negative posts reporting other complication, n (%)*
Instagram	15 (71.4)	2 (0.1)
Facebook	12 (21.8)	12 (21.8)
X	28 (87.5)	7 (21.9)

* Some negative posts were due to unspecified reasons, or reasons that were not due to pain or other complications.

**Table V.** Perceived outcome with concomitant procedure(s).

Concomitant procedure	Positive outcome (n)	Neutral outcome (n)	Negative outcome (n)
Hip scope	20	13	0
Femoral	2	1	0
Surgical hip dislocation	0	0	0
Second surgery following PAO	37	27	10
Other	49	42	6
Total	108	83	16

PAO, periacetabular osteotomy.

**Table VI.** Post popularity metrics by perceived outcome.

Social media platform	Perceived outcome	Mean number of likes (SD)	Mean number of comments (SD)	Mean number of shares (SD)
Instagram	Positive	58.8 (73.6)	6.2 (14.1)	-
	Neutral	37.6 (73.4)	3.8 (8.6)	-
	Negative	49 (17.1)	5.6 (3.4)	-
Facebook	Positive	22.1 (24.4)	10.4 (10.1)	-
	Neutral	2.3 (5.5)	11.4 (15.3)	-
	Negative	7.3 (9.3)	16.1 (15.5)	-
X	Positive	5.4 (8.8)	0.6 (1.3)	0.4 (1.1)
	Neutral	2.5 (7.1)	0.5 (1.9)	< 0.1 (0.2)
	Negative	1.9 (4.3)	0.5 (1.0)	0.2 (0.5)

## Discussion

Perceived experience data can be collected from social media platforms, providing valuable information for patients and surgeons.^7-12^ This is one of the largest retrospective case series to analyze perceived outcomes on social media and the first study, to our knowledge, focused on PAO.^13-21^ Prior literature has shown positive standardized patient-reported outcome measures in the PAO’s short-, intermediate-, and long-term timeframes; however, perceived outcomes may differ.^2-5,22^

This study provided data on the perceived outcomes posted on social media, and also aimed to identify factors associated with a negative perceived outcome. Patients who underwent PAO and subsequently posted content on social media regarding their perceived outcomes tended to be positive overall across all platforms. However, research has shown that social media users aim to convey a favourable self-image online.^23^Patients who reported any associated complication with their PAO were more likely to report a negative perceived outcome. In addition, if a negative perceived outcome was reported, this was more likely to be in the setting of having uncontrolled acute or chronic pain. This underscores the significance of engaging in preoperative discussions with patients regarding postoperative pain management strategies. In particular, it is especially crucial for surgeons who abstain from prescribing narcotics, given the backdrop of North America’s opioid crisis.^24^

Although studies have shown that patients with higher BMIs, higher Tonnis grades, older age, and prior hip arthroscopies are more likely to have surgical complications or failure, we could not stratify our data for this and see if this correlation also existed within our data.^4,22,25,26^ Furthermore, concomitant surgery with a PAO (i.e. arthroscopy, femoral osteotomy, bilateral PAO, or other additional surgery) was unrelated to increased negative perceived outcomes. This differed from prior findings, as studies have indicated an increased likelihood for negative early patient-reported outcomes in patients undergoing PAO in the setting of a previously failed hip arthroscopy.^22^ Additionally, although we could not stratify patient-perceived outcomes based on the duration of preoperative symptoms, studies have shown that the longer duration of symptoms (> two years) preoperatively does not significantly alter postoperative clinical outcome scores.^27^

Our findings suggest that perceived outcomes following PAO may vary based on the social media site used for data collection. Facebook PAO group members used social media to seek information. If group members reported outcomes, they trended towards neutrality and being informative. It should be noted that while we did not assess the validity of informational responses posted by patients on social media, prior literature has suggested poor accuracy of these posts.^28-34^ Instagram posts emphasized the outcomes of PAO, which were largely expressed through rehabilitation milestones and tended to be positive. Through our analysis, X users still tended to post about their positive perceived outcome with the PAO; however, this social media platform had the highest proportion of negative perceived outcomes. The variability of perceived outcomes between social media sites is crucial to mention, as patients and surgeons may not have a fully informed perception of the PAO if just one social media site is used to seek information.^35^ Despite the variability of perceived outcomes, most patients who post on social media had positive perceived outcomes regarding their PAO.

This study is limited by its retrospective nature and inclusion of English-language only posts. Additionally, the subjectivity of perception of PAO outcomes may not be directly related to the surgery itself. Our study is also limited by the lack of control (i.e. traditional surveys, standardized measurements of hip, function/symptom scores).^36,37^ While our study contains 2,559 posts, the sample size might not reflect the true PAO general population, since not everyone who undergoes a PAO procedure posts about their experience, uses social media in general, or implements hashtags with their posted content, which creates a selection bias. Additionally, the demographics and engagement vary among platforms, which inherently creates a reporting bias and variability among platforms in their own right. For example, Facebook is the most commonly used social media site with over 1.86 billion users in the USA, and one of most highly engaged with 70% of its users engaging daily. However, Facebook’s demographics include only 47% of users aged between 18 and 34 years – the primary age range for patients undergoing PAO. Furthermore, there may be other private groups that we could not access with the most relevant hashtags.

While the literature has shown good surgical, functional, and subjective outcomes associated with PAO surgery, perceived outcomes on social media sites may influence patients’ decisions to pursue surgery.^1-5^ With an increasingly higher number of patients using social media as a source of information, sometimes before speaking with their surgeon, common concerns regarding perceived outcomes can be addressed on the patient’s first surgical evaluation encounter. Surgeons can use this information to partner with patients to collaboratively make informed decisions and set expectations.^35^

Prior studies have shown that most patients use Google for surgeon selection, but many still use social media to help aid their decision.^38^ Although patients need to be able to express their own opinions, this highlights the need for surgeons to mitigate potential false narratives online so that patients can make a non-biased, informed decision. One potential solution would be for surgeons to accurately reflect their credentials and display real-time patient-reported outcome statistics through patient survey data on their social media profiles.^39^ However, this solution is limited by the relatively under-represented physician presence on social media, although orthopaedic surgery has a large social media presence among patients and hospitals.^19,40,41^ For surgeons who do not wish to maintain a social media presence, resources from the surgeons, including educational materials, support staff conversations, and (most importantly) adequate time for discussion, can potentially make a difference.

In conclusion, at a median of 84 days after PAO, the majority of posts collected on social media with content regarding a perceived outcome were positive. Although the overall proportion of positive, negative, and neutral posts varied between social media sites, all platforms reported more positive posts when compared to negative posts. Two factors were identified as predictive of a negative perceived outcome: 1) uncontrolled pain (acute or chronic); and 2) any additional surgical complication (nonunion, infection, secondary fracture, etc). Although patients sustaining these predictors were more likely to post a negative perceived outcome, the majority of these posts still tended to have a neutral perceived outcome across all social media platforms. These findings highlight the importance of considering the physical and psychosocial burdens of undergoing PAO, as they can have a major impact on the patient and their family.^42^ These findings emphasize the necessity of further research to define complications and address common questions raised on social media within this patient population.

**Take home message**

- This study highlights using social media as a resource for patients to share their perceived outcomes following periacetabular osteotomy (PAO).

- With the majority of dissatisfaction being attributed to postoperative pain, this emphasizes the need for providers to address and manage expectations and pain effectively in PAO patients, in order to enhance their overall satisfaction.

## Data Availability

The data that support the findings for this study are available to other researchers from the corresponding author upon reasonable request.
